# The ratio of fibroin to sericin in the middle silk gland of *Bombyx mori* and its correlation with the extensional behavior of the silk dope

**DOI:** 10.1002/pro.4907

**Published:** 2024-02-21

**Authors:** Teemu Välisalmi, Markus B. Linder

**Affiliations:** ^1^ Department of Bioproducts and Biosystems School of Chemical Engineering, Aalto University Aalto Finland; ^2^ Centre of Excellence in Life‐Inspired Hybrid Materials (LIBER) Aalto University Aalto Finland

**Keywords:** amino acid analysis, *Bombyx mori*, fibroin, middle silk gland, sericin

## Abstract

Understanding how native silk spinning occurs is crucial for designing artificial spinning systems. One often overlooked factor in *Bombyx mori* is the secretion of sericin proteins. Herein, we investigate the variation in amino acid content at different locations in the middle silk gland (MSG) of *B. mori*. This variation corresponds to an increase in sericin content when moving towards the anterior region of the MSG, while the posterior region predominantly contains fibroin. We estimate the mass ratio of sericin to fibroin to be ~25/75 wt% in the anterior MSG, depending on the fitting method. Then, we demonstrate that the improvement in the extensional behavior of the silk dope in the MSG correlates with the increase in sericin content. The addition of sericin may decrease the viscosity of the silk dope, a factor associated with an increase in the spinnability of silk. We further discuss whether this effect could also result from other known physicochemical changes within the MSG.

## 1 INTRODUCTION

A variety of animals spin silk with properties that exceed current human‐made materials (Heim et al., 2009; Koeppel & Holland, 2017). However, only the silkworm *Bombyx mori* can be employed to produce silk at a commercial scale (Koeppel & Holland, 2017). Other animal silks, including various types of spider silks, require recombinant production and artificial spinning to cost‐effectively generate silk fibers in sufficient quantities (Heim et al., 2009). Over the last decade, recombinant production of silk proteins has seen significant improvement, with certain silk proteins demonstrated to be producible at a feasible level (Saric & Scheibel, 2019; Schmuck et al., 2021). The methods of artificial spinning have also improved, with the most promising results achieved by imitating native spinning methods (Andersson et al., 2017; Arndt et al., 2022; Koeppel & Holland, 2017). Thus, a better understanding of native spinning may be key to achieving artificial silk fibers that match the properties of their native counterparts.

Native spinning of silk takes place in specialized silk glands (Andersson et al., 2016; Koeppel & Holland, 2017; Sutherland et al., 2010). The silk proteins of *B. mori*, fibroins, are produced at the beginning of the silk gland (posterior silk gland [PSG]; Andersson et al., 2016; Koeppel & Holland, 2017; Kundu et al., 2008). There are two different fibroin proteins: fibroin heavy chain (Fib‐H; 350 kDa) and fibroin light chain (Fib‐L; 26 kDa), which are connected by a disulfide bond (Andersson et al., 2016; Inoue et al., 2000; Koeppel & Holland, 2017). Fib‐H and Fib‐L have been shown to be secreted together with a glycoprotein P25 (27–30 kDa) in a molar ratio of 6:6:1 (Inoue et al., 2000). This silk dope is stored in the middle silk gland (MSG) in a soluble form at a very high concentration (Andersson et al., 2016; Koeppel & Holland, 2017). Additionally, different types of sericin proteins are secreted in the MSG (Andersson et al., 2016; Chen et al., 2012a; Kundu et al., 2008; Takasu et al., 2010). The silk dope moves towards the end of the gland—to the anterior silk gland (ASG)—which is a narrowing spinning duct connected to the mouth of the silkworm (Andersson et al., 2016; Koeppel & Holland, 2017). In the ASG, the silk dope is subjected to several chemical and physical changes, including pH and metal ion gradients, dehydration, shear, and elongational flow (Andersson et al., 2016; Domigan et al., 2015; Koeppel & Holland, 2017; Zhou et al., 2005). These changes drive the transition of the soluble silk dope into an insoluble silk fiber (Andersson et al., 2016; Domigan et al., 2015; Koeppel & Holland, 2017). The flow field in the silk gland is likely caused by external pulling of the silk, which occurs when the silkworm moves its head back and forth while spinning (Andersson et al., 2016; Sparkes & Holland, 2017). Silkworms have evolved separately from spiders to spin silk (Andersson et al., 2016; Holland et al., 2006). Interestingly, spiders also possess a very similar silk‐spinning system (Andersson et al., 2016; Holland et al., 2006). Therefore, understanding one system may provide useful information for designing an artificial spinning system for other silk proteins as well.

The sericin proteins of *B. mori* may also play a role in the spinning of silk (Sparkes & Holland, 2018). Sericin and fibroin both exhibit shear‐thinning viscoelastic behavior, with sericin demonstrating significantly lower viscosity (Sparkes & Holland, 2018). Thus, sericin has been suggested to act as a lubricant during spinning (Sparkes & Holland, 2018). In the spun fiber, sericin may function as a bonding agent (Chen et al., 2012a, 2012b; Kundu et al., 2008). At least three sericin genes of *B. mori* have been identified, which are expressed in different regions of the MSG (Du et al., 2011; Takasu et al., 2010). The mass ratio of sericin to fibroin has been reported to be 25/75% in commercial white cocoons (Cao & Zhang, 2016), while less is known about the silk dope in the MSG.

In this study, we report the amino acid composition of the MSG of *B. mori* as a function of position by employing amino acid analysis (AAA). We estimate the mass ratio between fibroin and sericin based on the amino acid content. We then demonstrate the extensionability of the silk dope from different parts of the MSG, showing that noticeable differences are visible already prior to the ASG.

## 2 MATERIALS AND METHODS

### 2.1 Silkworm dissection and native silk preparation

**FIGURE 1:**
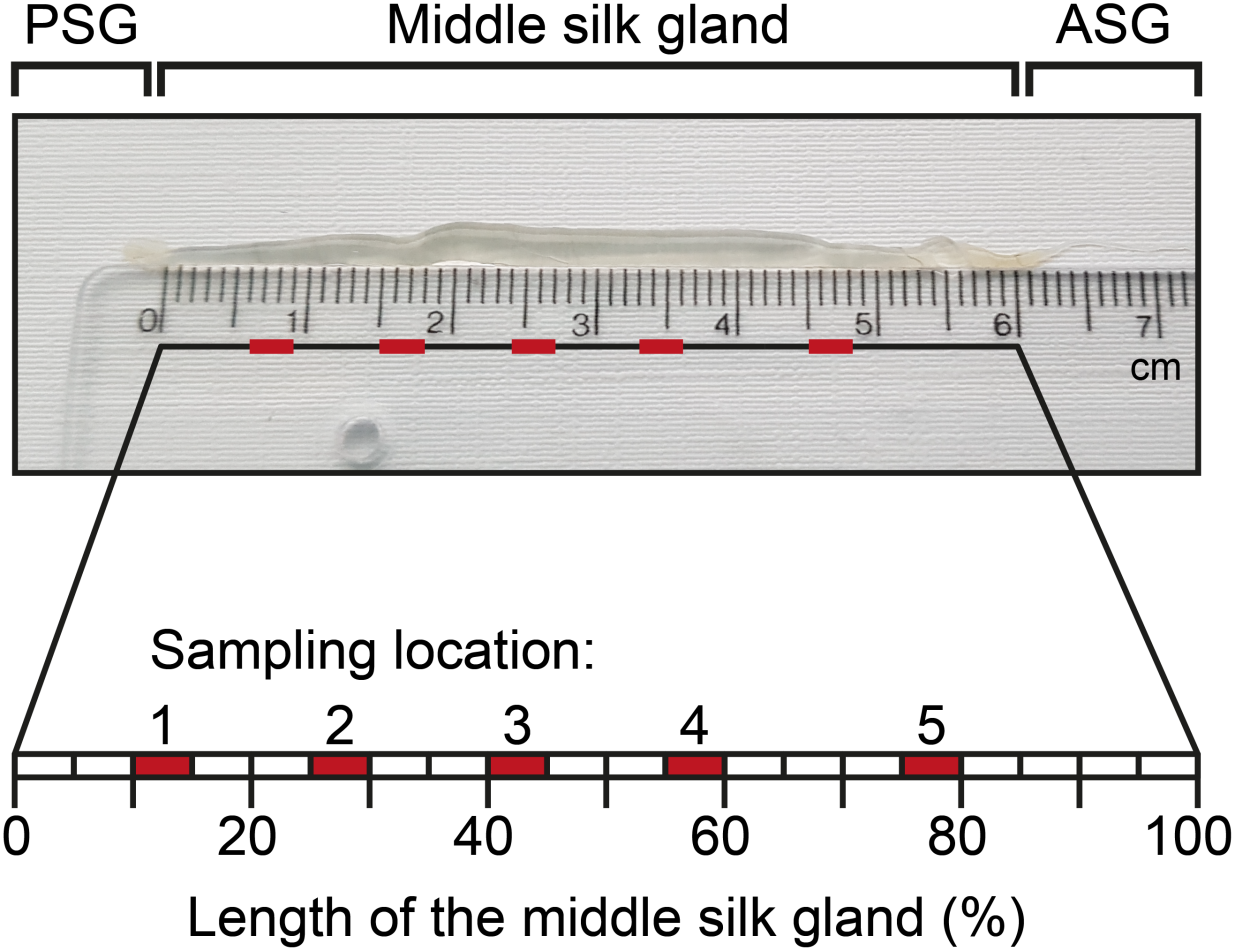
Sampling locations in the middle silk gland (MSG) are numbered 1–5. For example, Sample 1 is taken at ~10%–15% of the total length of the MSG. Thus, if the total length of the middle gland is 6 cm, Sample 1 is taken within 0.6–0.9 cm from the start of the MSG. ASG, anterior silk gland; PSG, posterior silk gland.

Native silk samples were gathered from silkworms (*B. mori*) at the end of the fifth instar, at the stage where they began constructing their silk cocoons. The silkworms were dissected by quickly cutting off the head and gently pushing the silk glands out. The glands were washed in ice‐cold distilled water, and excess water was removed with a lint‐free paper towel. The MSG region of the gland was cut in the locations numbered from 1 to 5, as shown in Figure 1. The contents of the gland were allowed to flow out without applying any additional pressure. Small silk dope samples (5–10 mg) were collected from the cutting locations. Any remaining epithelial layer was removed carefully. Silk dope samples were stored at 4°C for the AAA for a maximum of 3 days. Freshly collected silk dope samples were employed for the sodium dodecyl sulfate polyacrylamide gel electrophoresis (SDS‐PAGE) and extension experiments.

### 2.2 SDS‐PAGE

SDS‐PAGE was employed to separate and detect proteins in the native silk dope. Freshly collected silk dope samples were weighed (average weight 6.9 mg ± SD 1.1 mg) and mixed with SDS‐PAGE sample buffer (50 mM Tris–HCl pH 6.8, ~1 wt% SDS [0.16 g/mL], ~0.002 wt% bromophenol blue, 50 mM 1,4‐dithiothreitol [DTT], 10 vol% glycerol) in a ratio of 20 μL buffer per 1 mg of native silk dope. The mixtures were incubated for 2 h at room temperature and centrifuged for 5 min at 5000 rcf. Then, 10 μL of the supernatant was collected and incubated for 5 min at 95°C to denature the proteins. The samples were loaded onto a 4%–20% gradient precast polyacrylamide gel (Bio‐Rad no. 4568096), run at 100 V, and analyzed with a standard SDS‐PAGE procedure.

### 2.3 Amino acid analysis

AAA was employed to determine the total amino acid content of the native silk dope samples. The collected silk dope samples were weighed to measure the wet weight, then dried at 40°C, and reweighed to measure the dry weight. Dry silk dope samples were mixed with 1 mL of 6 M HCl containing 0.1 vol% phenol and 25 μL of 10 mM norleucine. Norleucine was employed as an internal standard. The samples were pipetted into vacuum hydrolysis tubes (ThermoFisher no. 29571), flushed three times with nitrogen to remove oxygen, and hydrolyzed at 110°C for 24 h under a nitrogen atmosphere. The HCl was evaporated under a vacuum (10 mbar) at 50°C. The remaining solid fraction was dissolved in 1 mL of sodium citrate buffer (pH 3.45) and filtered with a 0.2 μm hydrophilic polypropylene filter to remove any remaining particles. The total amino acid content of the filtrate was measured with a S433 Amino Acid Analyzer (Sykam GmbH, Germany). Elution was performed with a gradient of two sodium citrate buffers: buffer A with pH 3.45 and buffer B with pH 10.85. After elution, the amino acids reacted with ninhydrin at 130°C, forming derivates that were measured with a UV detector; 440 nm wavelength for proline derivate and 570 nm for derivates of other amino acids. Norleucine was employed to quantify the amount of each amino acid. Further details on calculating the amount of two different proteins or protein complexes in a sample are provided in Supporting information S1.

### 2.4 Pulling of silk

The extension of the silk dope from different locations of the silk gland was demonstrated with a motorized system and pulling by hand with two pairs of tweezers. The motorized system (Figure S1) was employed for an accurate control of the extension speed. The freshly collected silk dope (weight 5 ± 2 mg) was placed between two metal plates (diameter of 5.00 mm). The upper plate was attached to a motor drive train, allowing a steady extensional movement up to 1 mm/s. The silk dope was squeezed between the plates and then extended at a speed of 0.5 mm/s to a maximum length of 30 mm. The extension was also performed by pulling the silk dope with tweezers, which allowed a better grip of the silk dope. The freshly collected silk dope was placed between the tips of the tweezers and slowly pulled in opposite directions by hand until a maximum length of 10 cm. Both pulling experiments were performed at 53%–56% relative humidity (RH) and 20.1–20.6°C temperature.

## 3 RESULTS AND DISCUSSION

**FIGURE 2:**
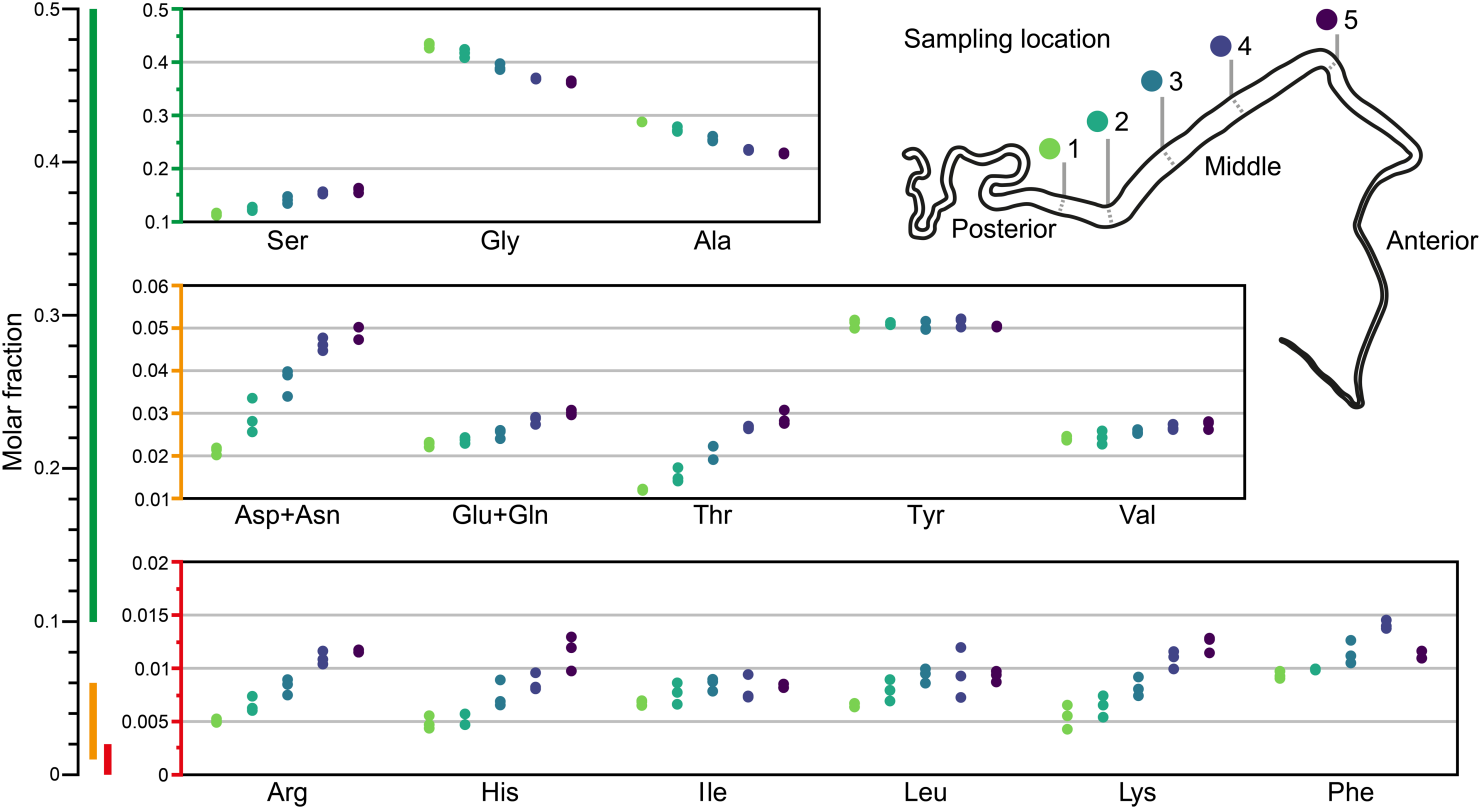
Amino acid content in the middle silk gland (MSG) after acid hydrolysis. Molar fractions of glycine and alanine decreased towards the end of the MSG, whereas most other amino acids increased by varying degrees. The amount of proline, cysteine, and methionine was below the detection limit.

Silk dope samples were collected from five locations along the MSG. The silk dope samples were weighed (average wet weight 8.42 mg ± SD 1.70 mg), then dried at 40°C, and reweighed (average dry weight 2.39 mg ± SD 0.49 mg). The silk dope samples were hydrolyzed, and the total amino acid content was measured (Figure 2). The molar fraction of alanine and glycine decreased from the start of the MSG (Location 1) to the end of the MSG (Location 5), while the molar fraction of serine, arginine, lysine, valine, glutamic acid/glutamine, threonine, isoleucine, leucine, histidine, and aspartic acid/asparagine increased to varying extents. Tyrosine and phenylalanine did not show any notable trend. Cysteine, methionine, and proline were below the detection limit and, therefore not included. The values for the amino acid content can be found in Table S1.

The amino acid content of the silk dope from the PSG and the posterior region of the MSG has previously been reported as 43–45 mol% alanine, 29–31 mol% glycine, 12 mol% serine, 4.8–5.2 mol% tyrosine, 1.6–2.4 mol% valine, and 5.9–7.3 mol% other amino acids (Shimura et al., 1976; Lucas et al., 1962; Sprague, 1975). These values align with our results from the posterior region of the MSG (Location 1), although we observed slightly higher values of certain amino acids—namely histidine, phenylalanine, aspartic acid (combined with asparagine), and glutamic acid (combined with glutamine)—and lower values of alanine. This discrepancy could be attributed to various factors, including the accuracy of the analytical measurements, or differences in the phenotype or rearing methods of *B. mori*. Similar experiments illustrating the trend in composition changes in the MSG have not been reported previously.

The total amino acid content in Location 1 closely aligns with the composition for Fib‐H, Fib‐L, and P25 in a molar ratio of 6:6:1 (Table 1 and Table S1). Likewise, the gradual shift in the amino acid content of the silk dope in the MSG mostly correlates with the increase in known and predicted sericin proteins: sericin 1 (Uniprot P07856), sericin 2 (D2WL76), and sericin 3 (A8CEQ1). The content of cysteine, methionine, and proline is low in the fibroin and sericin proteins, aligning with our results. The slight increase in valine suggests a higher amount of sericin 1 towards the anterior region. The increase of isoleucine could indicate the presence of either sericin 1 or 2. The absence of changes in tyrosine, however, suggests sericin 1. Additionally, the very low amount of proline in the samples indicates that sericin 2 is likely in a minor quantity. Thus, it is probable that the predominant portion of the sericin proteins is sericin 1 or its isoforms.

**TABLE 1:** Amino acid molar ratios of acid‐hydrolyzed fibroin complex and sericin proteins based on their amino acid sequences, and the expected and observed changes in amino acid content when moving towards the end of the MSG.

Amino acid	Fibroin complex	Sericin 1	Sericin 2	Sericin 3	Expected change	Observed change
Asp + Asn	0.015	0.129	0.188	0.120	Increase	Increase
Thr	0.010	0.089	0.059	0.028	Increase	Increase
Ser	0.120	0.327	0.151	0.436	Increase	Increase
Glu + Gln	0.011	0.045	0.135	0.118	Increase	Increase
Pro	0.004	0.007	0.056	0.002	‐	ND
Gly	0.443	0.110	0.043	0.120	Decrease	Decrease
Ala	0.295	0.072	0.034	0.052	Decrease	Decrease
Cys	0.001	0.004	0.001	0.004	‐	ND
Val	0.020	0.036	0.026	0.007	‐	Minor increase
Met	0.001	0.000	0.001	0.001	‐	ND
Ile	0.006	0.012	0.007	0.002	‐	Minor increase
Leu	0.005	0.019	0.015	0.006	Increase	Minor increase
Tyr	0.052	0.043	0.020	0.004	Decrease	No change
Phe	0.007	0.007	0.010	0.004	‐	No change
His	0.002	0.014	0.018	0.008	Increase	Increase
Lys	0.003	0.038	0.172	0.062	Increase	Increase
Arg	0.005	0.048	0.064	0.027	Increase	Increase

Abbreviations: MSG, middle silk gland; ND, not detected.

**FIGURE 3:**
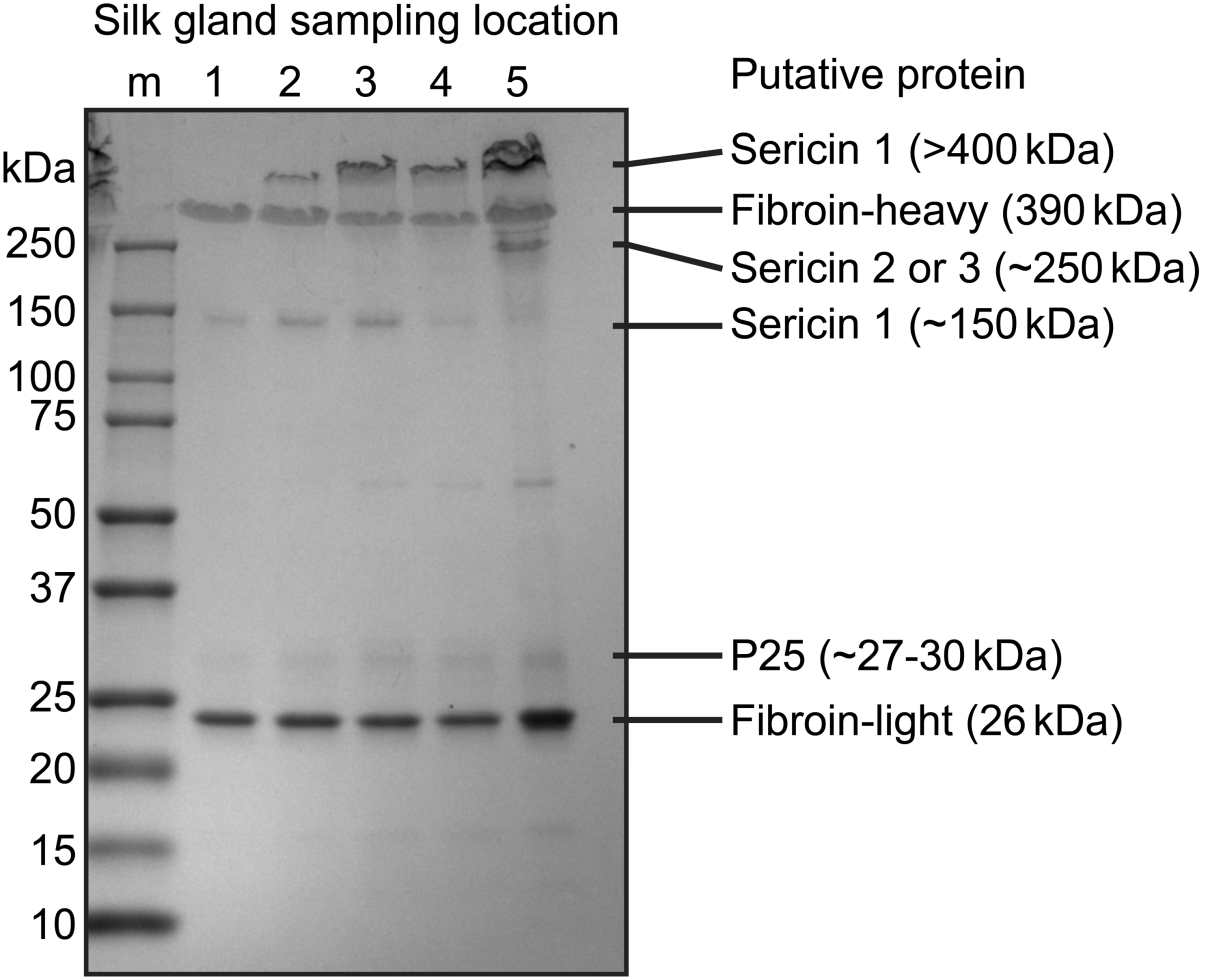
SDS‐PAGE of the silk dope samples from Locations 1 to 5. Proteins were identified based on literature (Takasu et al., 2002, 2010; Yamamoto et al., 2005). The results indicate a low concentration of high molecular weight (>400 kDa) protein, likely sericin 1, in Location 1, with an increase observed moving towards Location 5. Another isoform of sericin 1 may be observed at ~150 kDa, already secreted in Location 1. Secretion of sericin 2 or 3 becomes evident in Location 5. The molecular weight ladder is denoted as “m”.

Next, samples from the MSG Locations 1–5 were dissolved in SDS‐PAGE buffer containing DTT and analyzed with SDS‐PAGE (Figure 3). Repetitions of the experiment from eight different silkworms at the end of the fifth instar can be observed in Figure S2. All samples show two clear bands, likely corresponding to Fib‐H (390 kDa) and Fib‐L (26 kDa). The faint band above the 25 kDa mark aligns with glycoprotein P25, known to range from 27 to 30 kDa (Yamamoto et al., 2005). The band above Fib‐H corresponds to sericin 1, and the 250 kDa band may be either sericin 2 or 3 (Takasu et al., 2010). The faint band at ~150 kDa corresponds to “Sericin‐P,” an isoform of sericin 1 (Takasu et al., 2002). Consequently, the results indicate a low concentration of sericin in the posterior region of the MSG. Presumably, sericin 1 is secreted throughout the MSG, while secretion of sericin 2 or 3 initiates near the end of the MSG. These findings are in line with the amino acid results.

The secretion of ~400 and ~150 kDa sericin 1 isoforms has been reported to initiate in the middle and posterior regions of the MSG, respectively, while sericin 2 and 3 are reported to be present in the anterior region of the MSG (Takasu et al., 2007, 2010), consistent with our findings. However, the observed molecular weights of the putative sericin proteins do not match their estimated sizes in the Uniprot protein database. Sericin 1 and 2 have been reported to undergo splicing (Takasu et al., 2002, 2010), although the exact sequences of the various sericin isoforms are mostly unknown. Additionally, both fibroin and sericin proteins undergo posttranslational modifications, such as glycosylation, which further impacts their molar masses (Andersson et al., 2016; Chen et al., 2010; Du et al., 2011).

**FIGURE 4:**
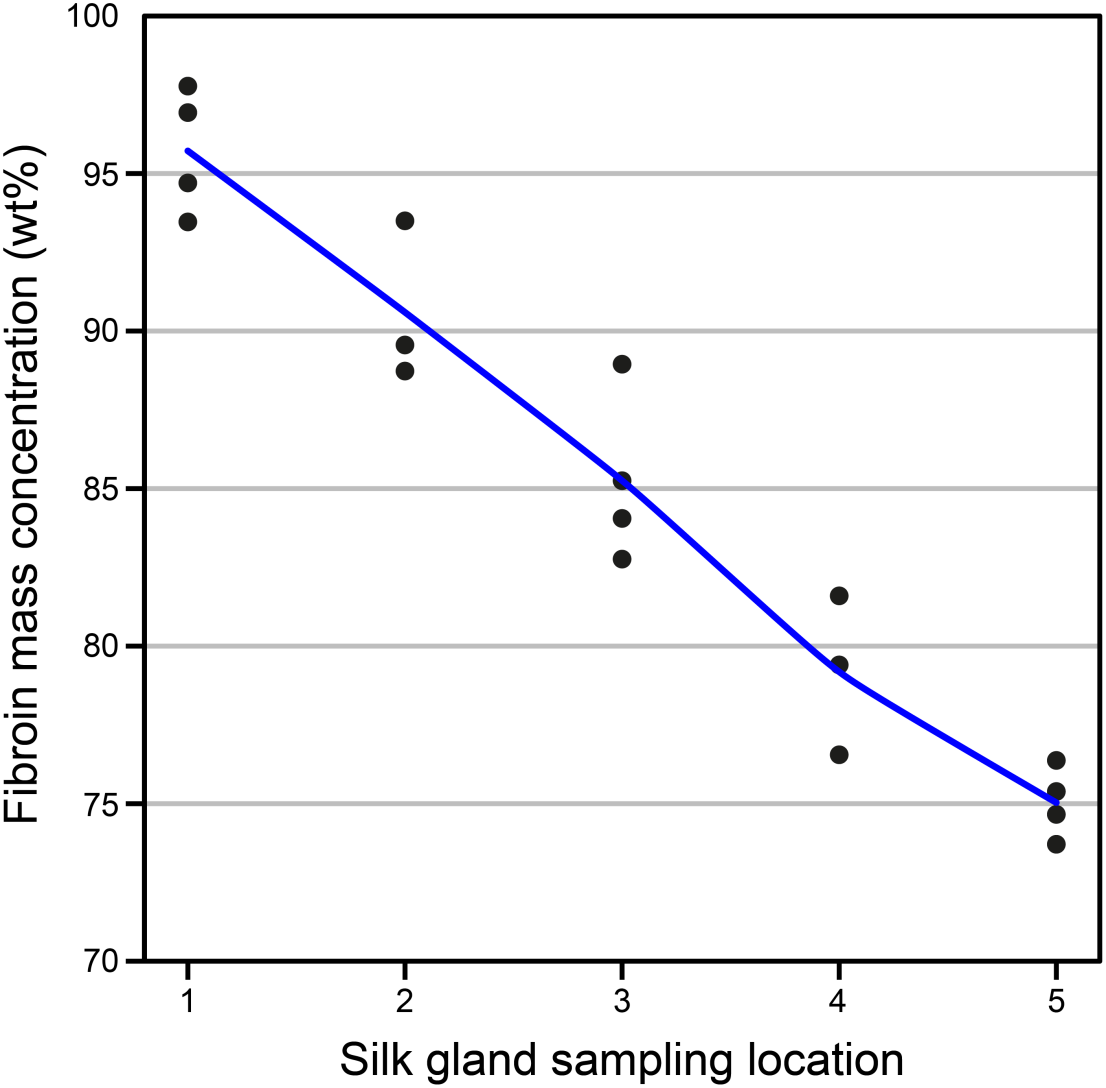
An estimation of the fibroin content in the total protein mass of silk dope samples, spanning from the start of the middle silk gland (Location 1) to the end of the middle silk gland (Location 5). The samples were measured with an amino acid analyzer, and the fibroin content was approximated under the assumption that the samples comprise of fibroin‐heavy, fibroin‐light, and P25 in 6:6:1 ratio (Inoue et al., 2000), along with sericin 1.

When the protein composition in the samples is limited to two distinct proteins or protein complexes, estimating the protein concentration in the sample becomes feasible through an iterative process, as elaborated in Supporting information S1. Assuming that the silk dope primarily comprises Fib‐H, Fib‐L, and P25 in molar ratio of 6:6:1, alongside sericin 1, the fibroin protein fraction of the total protein mass at the beginning of the MSG was found to be ~95 wt% (Figure 4). The concentration gradually decreased to 75 wt% by the end of the MSG. The coefficient of variation for the fits ranged from 0.15 to 0.2, whereas <0.05 would be expected for a pure mix of two proteins or protein complexes (Table S2 and Figure S3). Thus, it is evident that the MSG contains a considerable proportion of other proteins, and the findings should be regarded as estimations. Interestingly, substituting sericin 1 with sericin 2 or 3 resulted in a reduction in fibroin content from 97 to 84 and 95 to 82 wt%, respectively (Figures S4 and S5). However, in both instances, the coefficient of variation increased towards the end of the MSG, indicating a less accurate fit compared with sericin 1. This further supports the notion that the majority of the secreted sericin is likely to be sericin 1. Additionally, the amino acid content of the silk cocoons was measured. The fibroin content of the complete cocoon was estimated to be 77 wt%, with individual regions—floss, outer, middle, and inner cocoon layers—showing values of 65, 70, 83, and 82 wt%, respectively (Figures S6 and S7 and Table S3).

The composition of silk fibers across various *B. mori* strains has been reported to range from 65 to 85 wt% fibroin and 15–35 wt% sericin (Cao & Zhang, 2016; Kundu et al., 2008). Furthermore, commercially available white cocoons of *B. mori* have been measured to contain ~75 wt% fibroin and 25 wt% sericin (Cao & Zhang, 2016). These values closely align with our predictions for sericin and fibroin content in the anterior region of the MSG, indicating that the bulk of the sericin has been secreted at this point. This alignment is further supported by our prediction of fibroin and sericin contents in the freshly spun cocoons. However, it is important to note that our method of protein content estimation is indirect, and thus susceptible to error.

Approximately 93 wt% (± SD 3 wt%) of the silk dope dry weight was determined to be protein (Figure S8). No significant correlation was observed between the protein content and the MSG location (ANOVA *p*‐value 0.65). In experiments with pure bovine serum albumin, we found that, generally, 3–6 wt% of the protein was lost during hydrolysis, while internal standard norleucine remained intact (results not shown). Consequently, it is likely that the actual protein concentrations in the dried silk dope samples were closer to 95–100 wt%.

**FIGURE 5:**
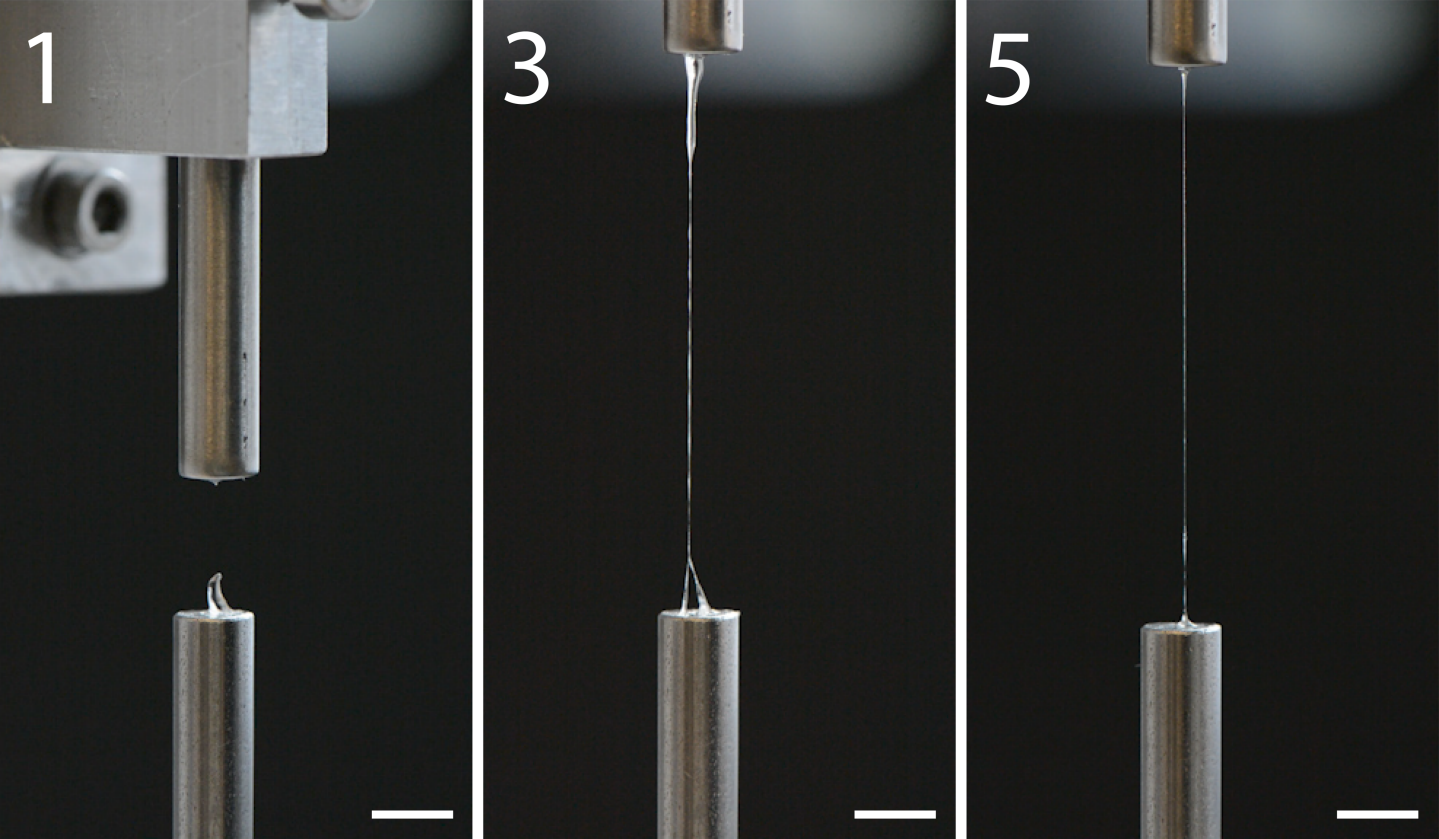
Extension of silk dope samples from the posterior (1), middle (3), and anterior (5) regions of the middle silk gland. The extension speed was maintained at 0.5 mm/s, with a total extension length reaching ~30 mm. Extension of silk dope Sample 1 was halted immediately upon necking‐induced breakage. The scale bar represents 5 mm. The complete video is available in Supporting information S1.

**FIGURE 6:**
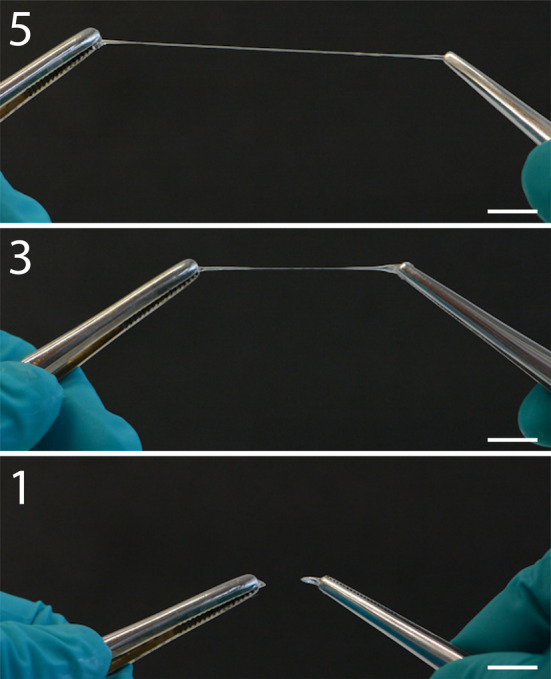
Extension by hand of silk dope samples from the posterior (1), middle (3), and anterior (5) regions of the middle silk gland. These snapshot images capture the silk dope samples at their maximum length, before experiencing a breakage. Samples from Locations 3 and 5 reached lengths of 40 and 70 mm, respectively. The sample from Location 1 broke quickly due to necking. The scale bar represents 10 mm. The complete video is available in Supporting information S1.

Next, silk dope samples were collected from MSG Locations 1, 3, and 5, and extended into filaments with a motorized setup. This method involved compressing a small piece of the silk dope between two metal plates and retracting the upper plate at a speed of 0.5 mm/s (Figure 5, Video S1, and Figure S9). With sufficient repetitions, it was possible to extend the silk dope from all locations into a filament; however, the likelihood of a successful extension increased notably towards the end of the MSG. Also, even with a very small sample amount, the silk dope from the end of the MSG could be extended into a full‐length filament (30 mm). Nevertheless, multiple other factors appeared to have a varying effect on the results, such as the precise way of cutting the silk dope sample with a scalpel, removal of excess water from the silk gland after washing, the exact volume of the silk dope sample, and the placement of the silk dope sample on the metal plate. Moreover, adhesion of the silk dope to the metal plates was inconsistent and did not seem to be dependent on the sample type. Comparable extension experiments were conducted by securing a silk dope sample between two pairs of tweezers and slowly pulling them in opposite directions (Figure 6 and Video S2). Similar to the motorized setup experiments, pulling with tweezers revealed that the silk dope from the end of the MSG was more readily extended into a filament. At the beginning of the pulling, the filaments appeared to fail due to necking, but as they lengthened, failure typically occurred due to elastic fracture.

The improvement in the extensional behavior of the silk dope correlates with an increase in sericin content. The increased sericin levels are likely to reduce the viscosity of the silk dope, which has been shown to improve the spinnability of the silk dope (Koeppel et al., 2020). Alongside the rise in sericin content, various physicochemical changes, including salts, pH, and dehydration, occur in the MSG. Notably, the pH in the silk gland transitions from 7 to 8 in the PSG, to ~7 in the MSG, further decreasing to 6.8 between the MSG and the ASG, and reaching 6.5–6.2 in the posterior region of the ASG (Domigan et al., 2015).

Within the pH range of 6–7, the terminal domains of the fibroin are suggested to initiate a transition of fibroin into β‐sheet rich structure, and induce oligomerization (Domigan et al., 2015). While these processes play a significant role in fiber formation, their impact is likely minor within the anterior region of the MSG. Additionally, the concentrations of ions K^+^, Mg^2^+, Cu^2+^, Zn^2+^, and Na^+^ have been reported to increase, and Ca^2+^ to decrease, from the posterior to the anterior region of the MSG (Zhou et al., 2005). The decrease of Ca^2+^ and increase of K^+^ have been associated with a reduction in the viscosity of the silk dope (Koeppel et al., 2020). Thus, it is not surprising that we observed the silk dope from the anterior MSG to be easier to extend into a filament.

Extensional behavior of the silk dope has been previously studied by Koeppel et al. (2018, 2021). They showed that the silk dope from the posterior region of the MSG can be extended into a 20 mm long filament, with extension rates ranging from 0.05 to 0.31/s (Koeppel et al., 2018). At higher rates, the filaments were more prone to break due to elastic failure (Koeppel et al., 2018), consistent with our results from extension by hand. They also demonstrated that dehydration at 55% RH was beneficial for filament formation compared with 98% RH (Koeppel et al., 2018). Our experiments were conducted at 55% RH. Furthermore, they showed that an acidic environment is beneficial for the filament formation (Koeppel et al., 2021), which is associated with the structural changes in fibroins induced by pH variations.

## 4 CONCLUSION

Here, we report a change in amino acid content when transitioning from the posterior to the anterior region of the MSG. These changes correspond to the secretion of different sericin proteins. Through comparisons with previous reports, the emergence of the ~400 kDa isoform of sericin 1 is evident after the first quarter of the MSG, the ~150 kDa isoform of sericin 1 is noticeable at the start of the MSG, and the ~250 kDa sericin (2 or 3) is observed at the end of the MSG. Overall, the majority of the secreted sericin likely comprises isoforms of sericin 1. With an indirect model based on amino acid composition, we estimated that the fibroin content at the start of the MSG is 95–97 wt% and gradually decreases to 75–84 wt% at the end of the MSG, depending on the fitting method, with the remainder being sericin. Furthermore, we demonstrated that the silk dope from the anterior region of the MSG is easier to extend into a filament, indicating that physicochemical changes to the silk dope are already apparent within the MSG.

## AUTHOR CONTRIBUTIONS

**Teemu Välisalmi:** Conceptualization; investigation; writing—original draft; methodology; validation; visualization; formal analysis; data curation. **Markus B. Linder:** Conceptualization; funding acquisition; writing—review and editing; project administration; supervision; resources.

## FUNDING INFORMATION

This work was funded by the Academy of Finland project no. 317019, the Center of Excellence Program (2022–2029) in Life‐Inspired Hybrid Materials (LIBER) project no. 346105, and the Novo Nordisk Foundation (no. 0061306).

## CONFLICT OF INTEREST STATEMENT

The authors declare no competing financial interest.

## Supporting information


**FIGURE S1.** Motorized system to extend silk dope samples.
**FIGURE S2.** SDS‐PAGE of the silk dope samples from the middle silk gland from Locations 1–5.
**FIGURE S3.** Example of solving molar ratio of lysozyme of a sample containing 75 wt% of lysozyme and 25 wt% of BSA.
**FIGURE S4 and S5.** Fibroin content of the total protein mass in the silk dope samples with the assumption that they contain sericin 2 or 3, and Fib‐H, Fib‐L, and P25 in 6:6:1 ratio.
**FIGURE S6.** Fibroin content of silk cocoons.
**FIGURE S7.** Amino acid content of the different regions of silk cocoons.
**FIGURE S8.** Protein content of dried silk dope samples.
**FIGURE S9.** Extension of the silk dope from the posterior, middle, and anterior region of the middle silk gland using a motorized pulling setup.
**TABLE S1.** Mole fractions of the amino acids of the silk dope samples from the middle silk gland Locations 1–5.
**TABLE S2.** Samples containing lysozyme and BSA for testing amino acid analysis of complex protein samples.
**TABLE S3.** Molar fractions of the amino acids in the different regions of *B. mori* silk cocoon.

**Video S1.** Extensional pulling of silk dope samples with a motorized setup.

**Video S2.** Extensional pulling of silk dope samples by hand.
