# Supplementary material for: The ratio of fibroin to sericin in the middle silk gland of Bombyx mori and its correlation with the extensional behavior of the silk dope
Source: Protein Sci. 2024 Feb 21;33(3):e4907. doi: 10.1002/pro.4907 (PMC10880417; doi:10.1002/pro.4907)
Supplement: Supplementary file 1 — FIGURE S1. Motorized system to extend silk dope samples. FIGURE S2. SDS‐PAGE of the silk dope samples from the middle silk gland from Locations 1–5. FIGURE S3. Example of solving molar ratio of lysozyme of a sample containing 75 wt% of lysozyme and 25 wt% of BSA. FIGURE S4 and S5. Fibroin content of the total protein mass in the silk dope samples with the assumption that they contain sericin 2 or 3, and Fib‐H, Fib‐L, and P25 in 6:6:1 ratio. FIGURE S6. Fibroin content of silk cocoons. FIGURE S7. Amino acid content of the different regions of silk cocoons. FIGURE S8. Protein content of dried silk dope samples. FIGURE S9. Extension of the silk dope from the posterior, middle, and anterior region of the middle silk gland using a motorized pulling setup. TABLE S1. Mole fractions of the amino acids of the silk dope samples from the middle silk gland Locations 1–5. TABLE S2. Samples containing lysozyme and BSA for testing amino acid analysis of complex protein samples. TABLE S3. Molar fractions of the amino acids in the different regions of B. mori silk cocoon. [file PRO-33-e4907-s001.docx]

Supporting information

## **The ratio of fibroin to sericin in the middle silk gland of *Bombyx mori* and its correlation with the extensional behavior of the silk dope**

Teemu Välisalmi^1,2*^, Markus B. Linder^1,2*^

1: Department of Bioproducts and Biosystems, School of Chemical Engineering, Aalto University, FI-00076 Aalto, Finland

2: Centre of Excellence in Life-Inspired Hybrid Materials (LIBER), Aalto University, P.O. Box 16100, 00076 Aalto, Finland

Motorized pulling setup


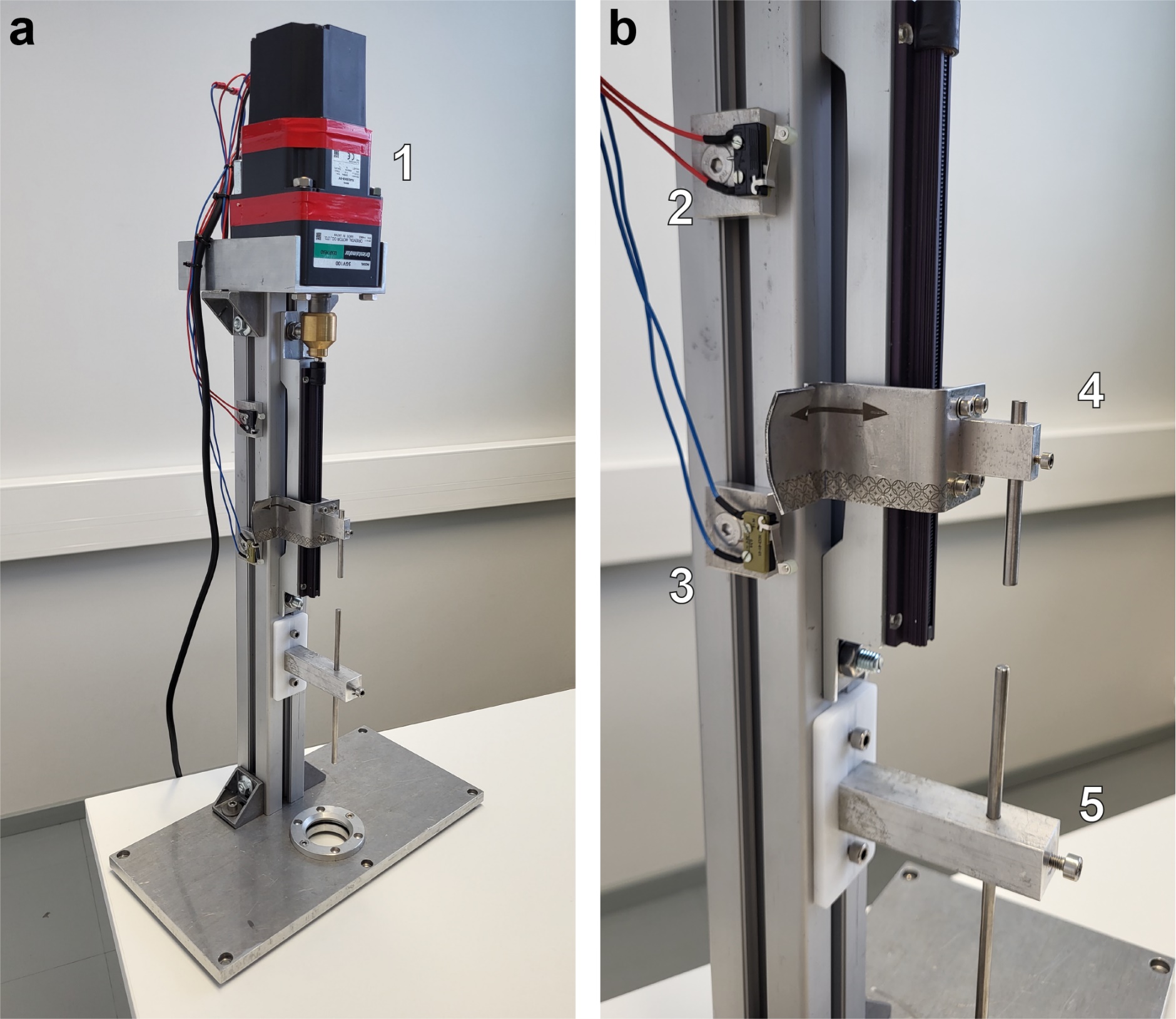


**Figure S1**. (a) Motorized setup to extend silk dope samples. Motor setup (1) consisted of Orientalmotor BLA550CB-GV (motor) and 5GV100 (parallel shaft gear head). (b) Zoomed view of the device. Upper (2) and lower (3) limit switches were installed to set a maximum and minimum distance for the moving stage (4). Lower stage (5) was fixed in place.

Amino acid content in middle silk gland samples

**Table S1**. Molar fractions of the amino acids in the middle silk gland (MSG) from location 1 (posterior MSG) to location 5 (anterior MSG). Proline, cysteine, and methionine were not detected. The reported values are averages of three samples.

|  | Molar fraction in MSG locations | | | | |
| --- | --- | --- | --- | --- | --- |
| Amino acid | 1 | 2 | 3 | 4 | 5 |
| Asp + Asn | 0.021 | 0.029 | 0.038 | 0.047 | 0.049 |
| Thr | 0.011 | 0.015 | 0.021 | 0.027 | 0.029 |
| Ser | 0.112 | 0.122 | 0.140 | 0.153 | 0.158 |
| Glu + Gln | 0.023 | 0.024 | 0.025 | 0.028 | 0.030 |
| Gly | 0.431 | 0.416 | 0.391 | 0.370 | 0.363 |
| Ala | 0.288 | 0.275 | 0.256 | 0.235 | 0.229 |
| Val | 0.024 | 0.024 | 0.025 | 0.027 | 0.027 |
| Ile | 0.007 | 0.008 | 0.008 | 0.008 | 0.008 |
| Leu | 0.007 | 0.008 | 0.009 | 0.010 | 0.009 |
| Tyr | 0.051 | 0.051 | 0.050 | 0.051 | 0.050 |
| Phe | 0.009 | 0.010 | 0.011 | 0.014 | 0.011 |
| His | 0.005 | 0.005 | 0.007 | 0.009 | 0.012 |
| Lys | 0.005 | 0.006 | 0.008 | 0.011 | 0.012 |
| Arg | 0.005 | 0.007 | 0.008 | 0.011 | 0.012 |

SDS-PAGE of silk dope samples from eight silkworms at the end of fifth instar


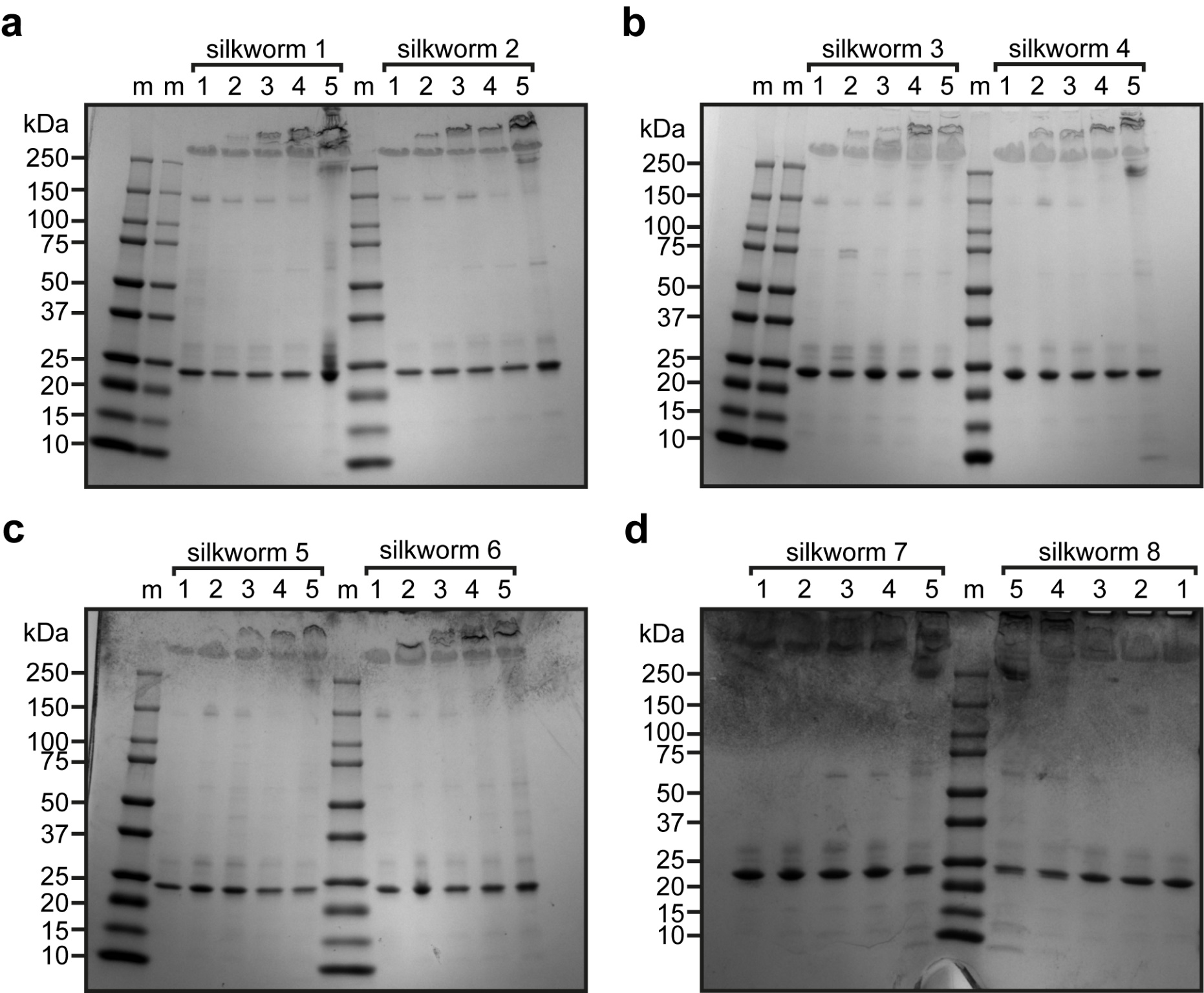


**Figure S2**. SDS-PAGE of the silk dope samples from the middle silk gland (MSG) from locations 1 (posterior of MSG) to 5 (anterior of MSG). The samples were taken from eight different silkworms at the end of fifth instar. The results are consistent with each other and show increased secretion of different sericin proteins when moving closer to the end of the MSG. Gels (c) and (d) contain blemishes due to contamination for unknown reason. The molecular weight ladder is marked as “m”. The gel “silkworm 2” is shown in Figure 3.

Amino acid analysis of complex samples

Amino acid analysis (AAA) was employed to quantify the amount of individual amino acids in the sample. If the sample is comprised of mostly one protein, the protein amount can be calculated from the amino acid composition [4]. However, this method does not work with more complex samples. An iteration is needed to quantify the amount of proteins in a sample with two or more different proteins. The concept was tested by preparing mixes of bovine serum albumin (BSA) and lysozyme, as shown in Table S2. The samples were hydrolyzed, and AAA was performed as described in Materials and Methods 2.3. The AAA results provides the substance amount (mass) for each amino acid. If the sample consists mostly of one protein, the mass of each amino acid is divided by the expected number of the amino acid in the protein sequence, and an average of the resulting values is taken to acquire the mass amount of that protein. For a sample consisting of two proteins (lysozyme and BSA), the protein sequence is replaced with an expected amino acid sequence, given by

$$S_{total}=x*S_{lysozyme}+\left( 1-x \right)*S_{BSA}$$

where $S_{total}$ is the expected amino acid sequence, $x$ is molar fraction of lysozyme, and $S_{lyzosyme}$ and $S_{BSA}$ are the amino acid sequences of lysozyme and BSA, respectively.

The expected amino acid sequence, $S_{total}$, was fitted to the measured amino acid sequence, and the accuracy of the fit was evaluated with coefficient of variation. An iteration was performed, where the molar fraction of lysozyme, $x$, was set as the variable, and coefficient of variation was set to be minimized (Figure S3). The total degradation of tryptophan, and conversion of glutamine and asparagine into glutamic acid and aspartic acid, respectively, caused by the acidic hydrolysis, were accounted for in the expected protein sequences. Also, cysteine and methionine were not considered in the calculation of the protein concentrations due to their substantial degradation during the hydrolysis.

**Table S2**. Samples containing lysozyme and BSA for testing amino acid analysis of complex protein samples. Purity of the used lysozyme and BSA extracts were >98% and >99.5%, respectively.

| Sample | Lysozyme (wt%) | BSA (wt%) | Calculated amount of lysozyme (mass ratio) | Coefficient of variation |
| --- | --- | --- | --- | --- |
| 100L | 100 | 0 | 0.969 | 0.047 |
| 75L | 75 | 25 | 0.725 | 0.040 |
| 50L | 50 | 50 | 0.474 | 0.050 |
| 25L | 25 | 75 | 0.222 | 0.036 |
| 0L | 0 | 100 | 0.008 | 0.036 |


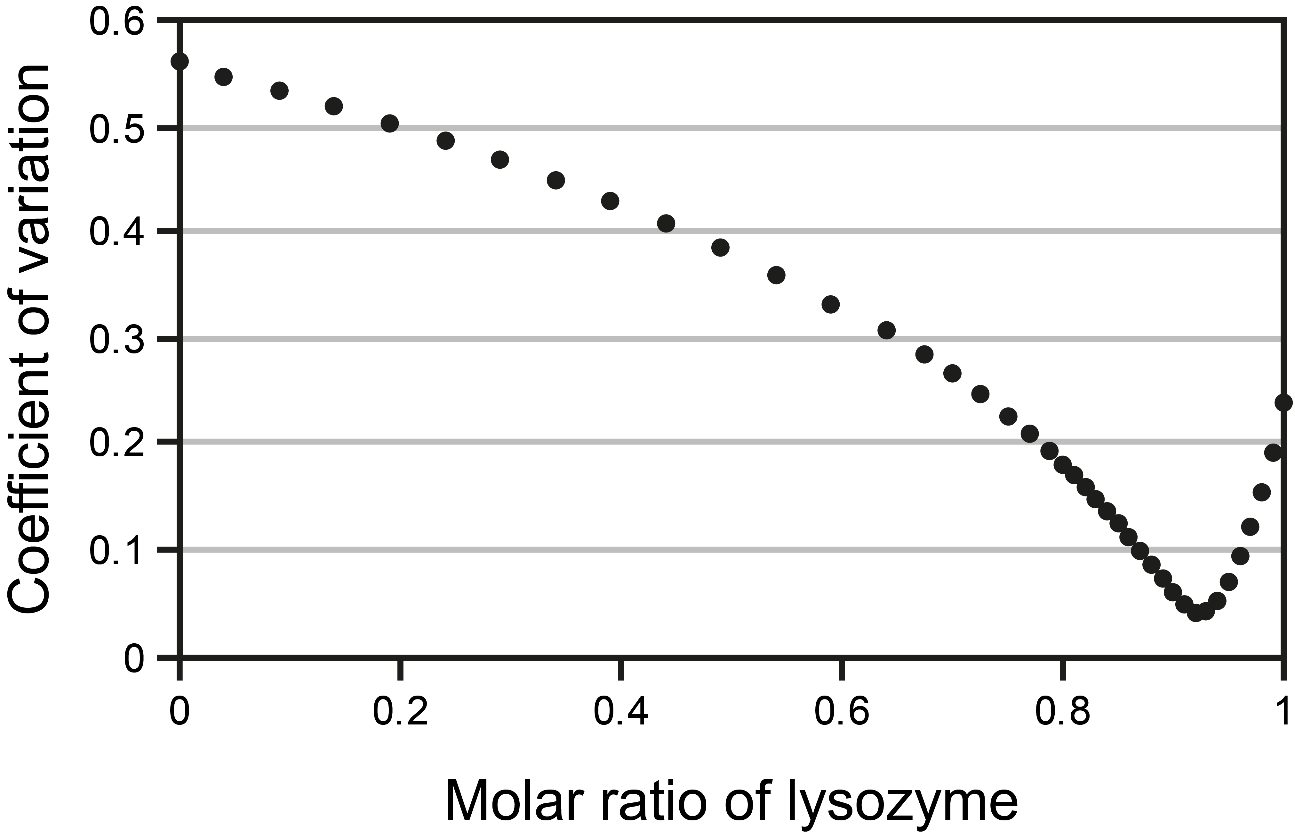


**Figure S3**. Example of solving molar ratio of lysozyme of a sample containing 75 wt% of lysozyme and 25 wt% of BSA (denoted as 75L). The coefficient of variation reached its minimum at a lysozyme molar ratio of 0.924, corresponding to a mass ratio of 0.725 (i.e., 72.5 wt% of lysozyme).

Estimation of the fibroin content in the silk dope samples from MSG


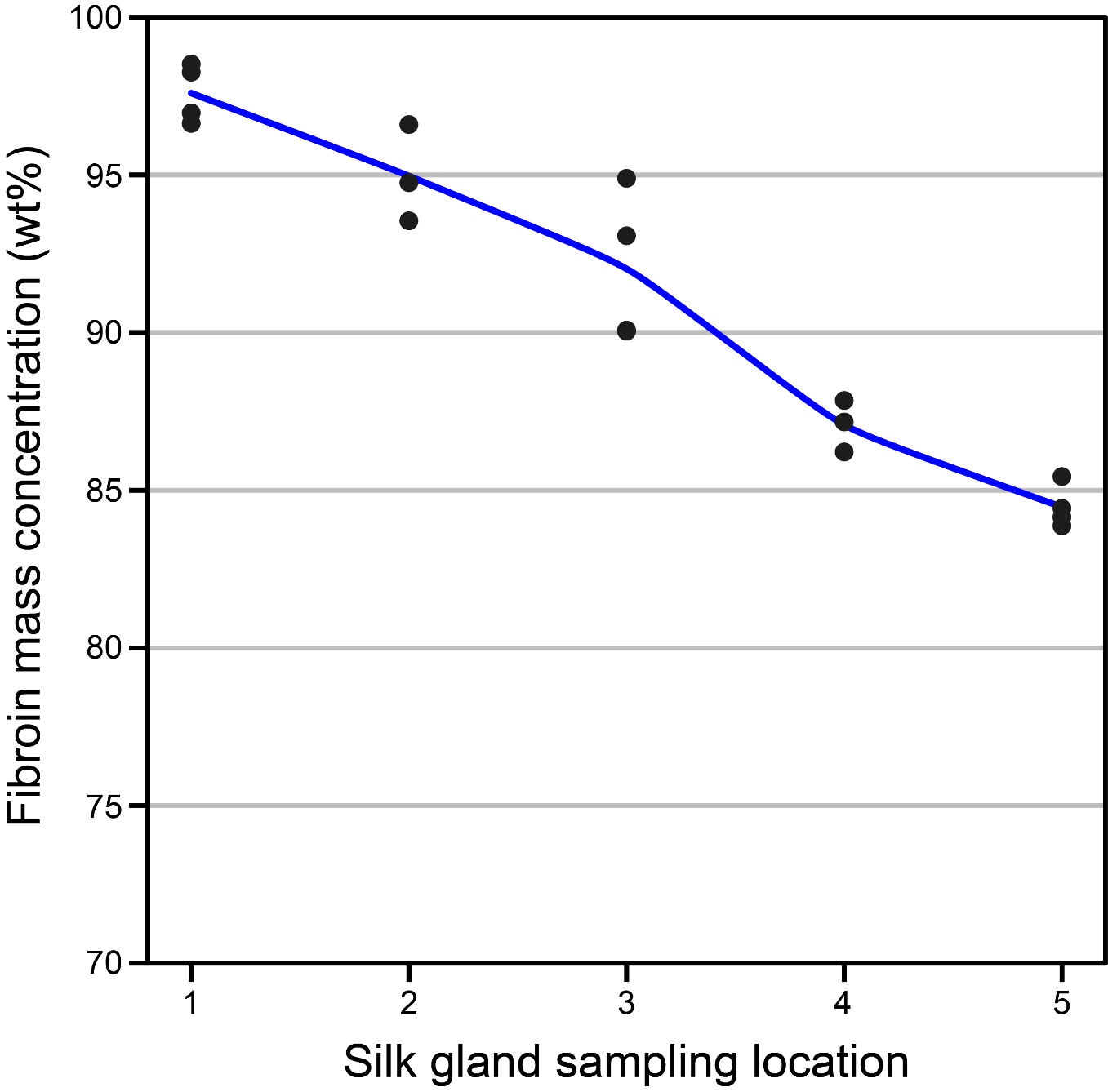


**Figure S4**. Fibroin content of the total protein mass in the silk dope samples, with the assumption that they contain sericin 2 (Uniprot D2WL76), and fibroin-heavy, fibroin-light, and glycoprotein P25 in 6:6:1 ratio [5]. Silk dope samples were taken from the start of the middle silk gland (location 1) to the end of the middle silk gland (location 5). The samples were hydrolyzed, and then measured with an amino acid analyzer.


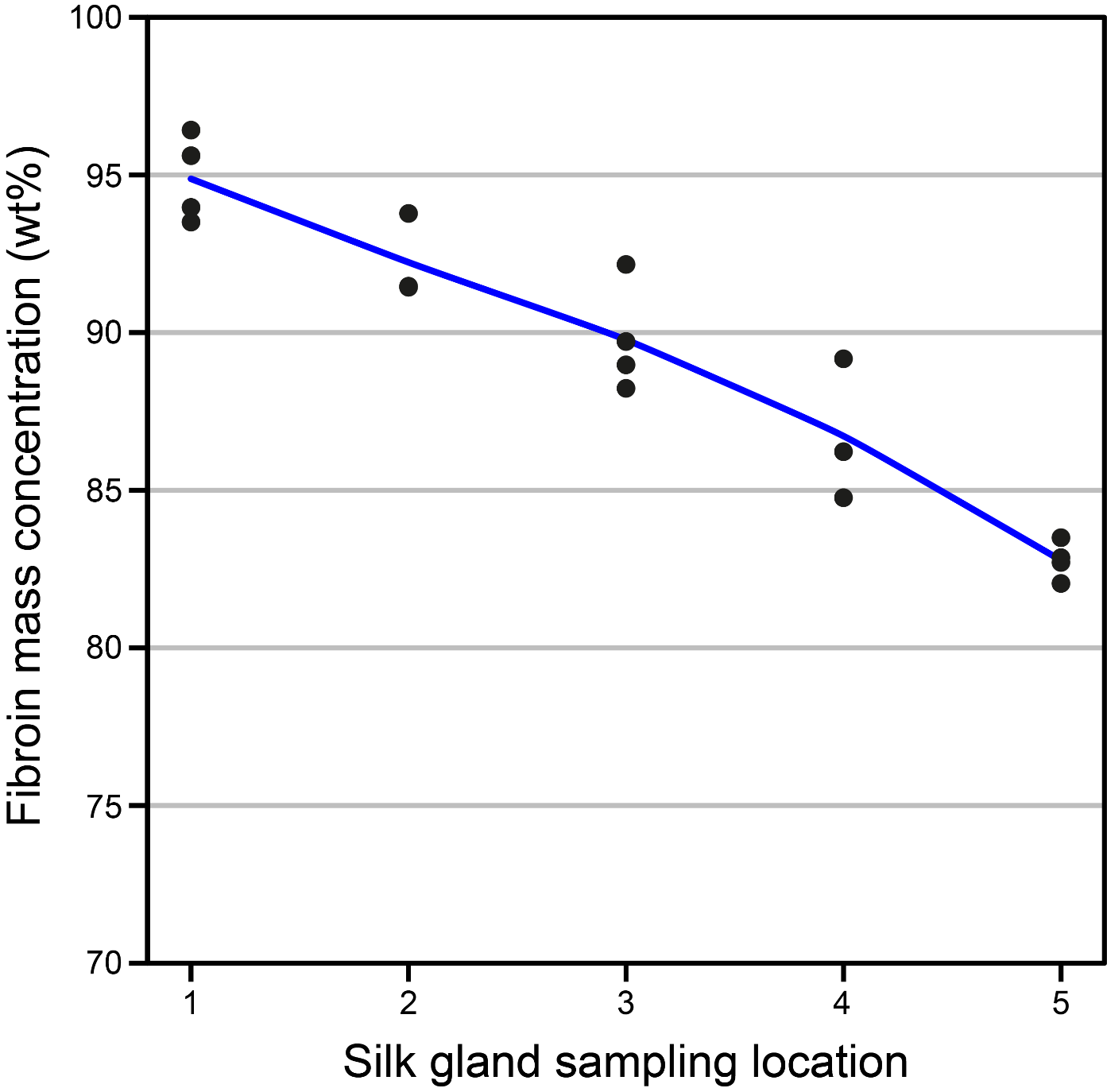


**Figure S5**. Fibroin content of the total protein mass in the silk dope samples, with the assumption that they contain sericin 3 (Uniprot A8CEQ1), and fibroin-heavy, fibroin-light, and glycoprotein P25 in 6:6:1 ratio [5]. Silk dope samples were taken from the start of the middle silk gland (location 1) to the end of the middle silk gland (location 5). The samples were hydrolyzed, and then measured with an amino acid analyzer.

Fibroin and amino acid content of silk cocoons

Freshly spun cocoons of *B. mori* were employed for the analysis. First, the soft floss of the cocoon was collected, and the remaining cocoon was divided into three equally thick layers: outer, middle, and inner layers (Figure S6a). Additionally, a radial cut of the complete cocoon, including the floss layer, was collected. The samples were dried at 60 °C overnight to remove moisture. The dried samples weighed 1.3 mg ± SD 0.21 mg. Samples were taken from three cocoons. The samples were hydrolyzed, and then measured with the amino acid analyzer (Materials and Methods 2.3). Fibroin content was estimated with the assumption that the samples are primarily comprised of Fib‑H, Fib-L, and P25 in molar ratio of 6:6:1, and sericin 1 (Uniprot P07856). The radial cut of the complete cocoon showed an average fibroin content of 76.9 wt%, and the floss, outer, middle, and inner layers showed 65.4, 69.5, 82.5, and 82.2 wt%, respectively (Figure S6b). The coefficient of variation for the fits were within 0.10–0.14, indicating a good fit. The amino acid content is shown in Figure S7 and Table S3.


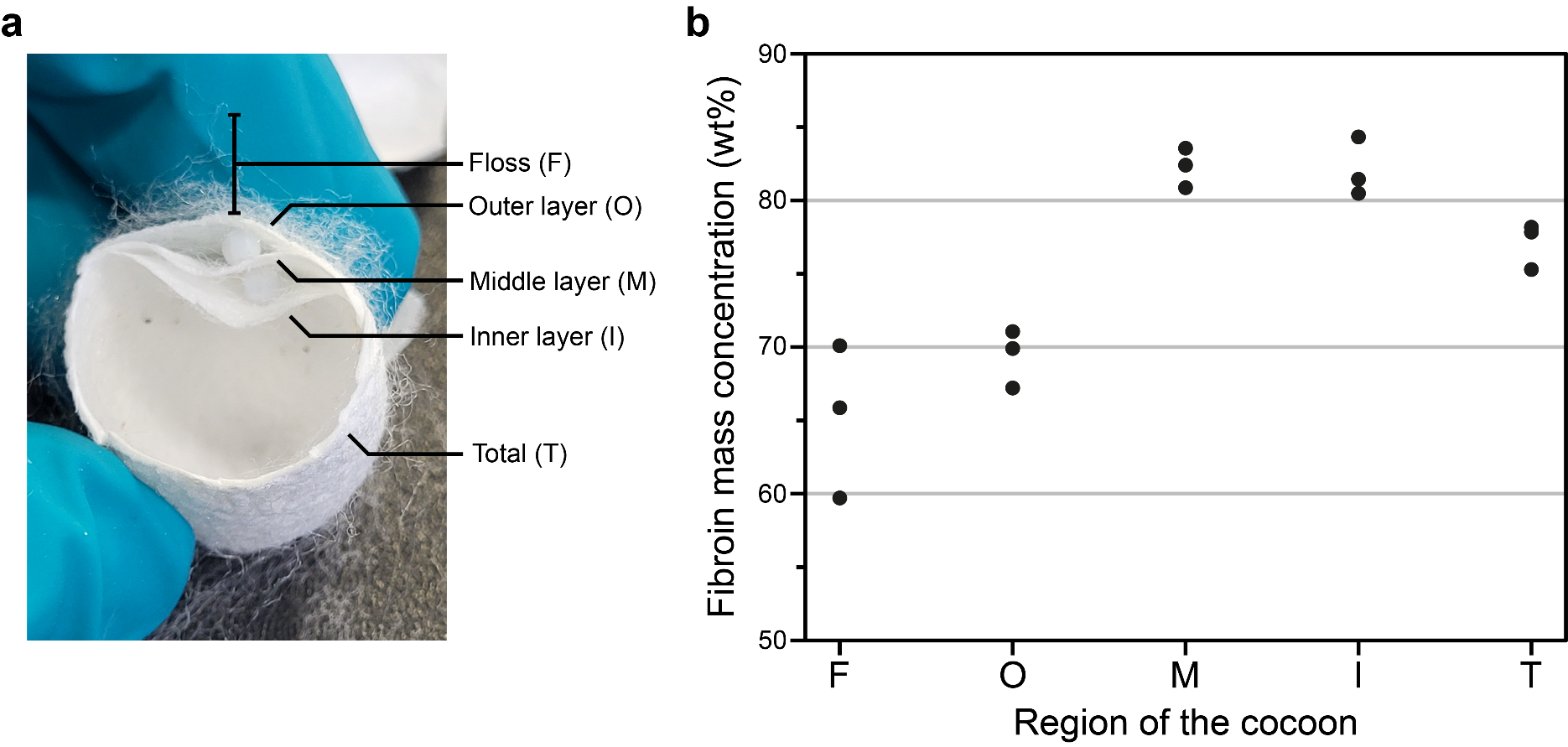


**Figure S6**. Fibroin content of *B. mori* silk cocoons. (a) Samples were collected from the floss, and three layers of the cocoon (outer, middle, inner). Additionally, a radial cut of the complete cocoon, including the floss layer, was collected. For visual clarity, the layers have been separated with two white spheres. (b) The samples were hydrolyzed, and then measured with an amino acid analyzer. The fibroin content of the total protein mass was estimated with the assumption that the samples contain sericin 1 (Uniprot P07856), together with fibroin-heavy, fibroin-light, and glycoprotein P25 in 6:6:1 ratio [5].


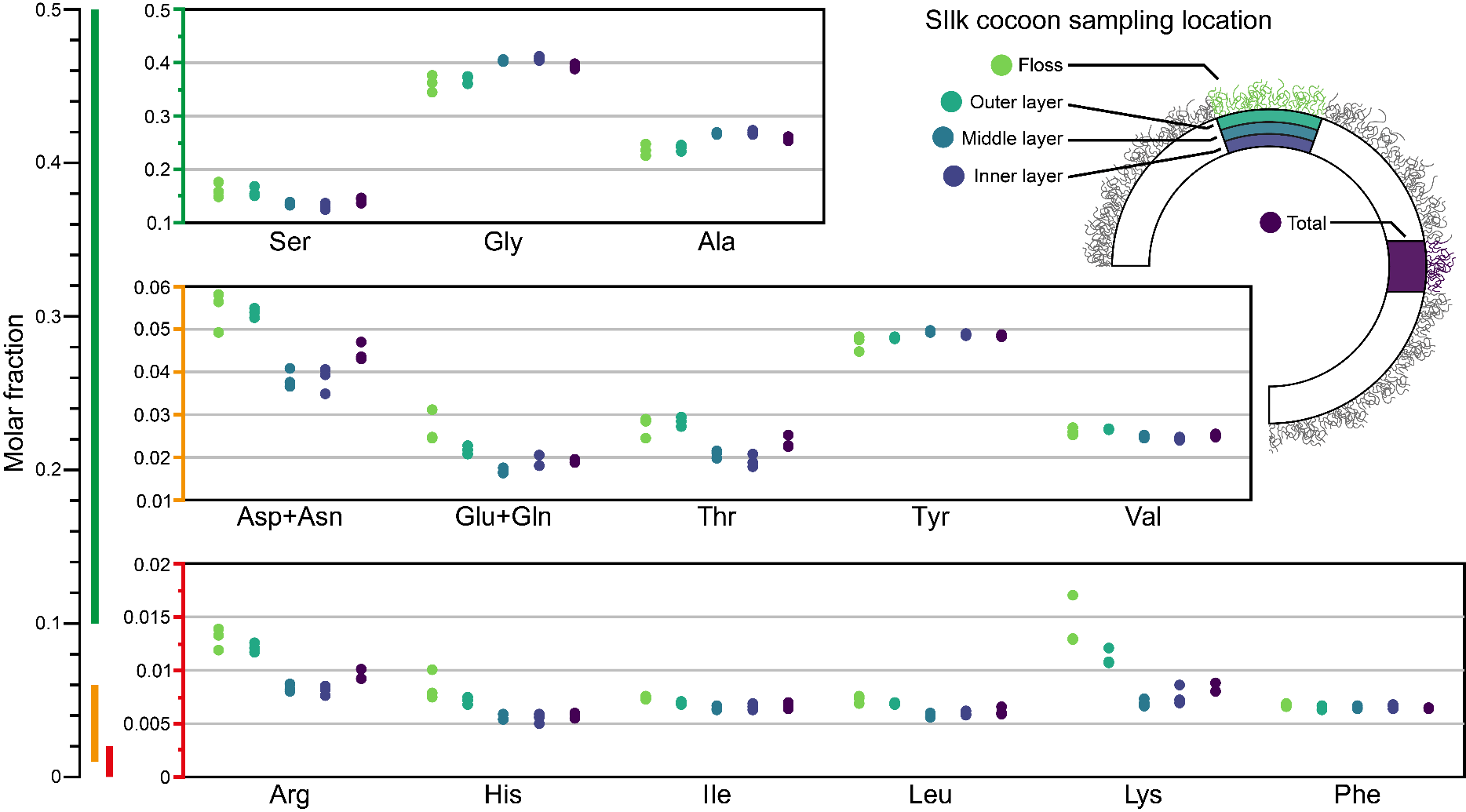


**Figure S7**. Amino acid content of the different regions of *B. mori* silk cocoon after acidic hydrolysis. Proline, cysteine, and methionine were not included.

**Table S3**. Amino acid molar fraction of the different regions of *B. mori* silk cocoon after acidic hydrolysis. Proline, cysteine, and methionine were not included. The reported values are averages of three samples.

|  | Molar fraction in different regions of silk cocoon | | | | |
| --- | --- | --- | --- | --- | --- |
| Amino acid | Floss | Outer layer | Middle layer | Inner layer | Total |
| Asp + Asn | 0.055 | 0.054 | 0.039 | 0.038 | 0.045 |
| Thr | 0.027 | 0.028 | 0.021 | 0.019 | 0.023 |
| Ser | 0.161 | 0.158 | 0.136 | 0.131 | 0.143 |
| Glu + Gln | 0.027 | 0.022 | 0.017 | 0.019 | 0.019 |
| Gly | 0.362 | 0.371 | 0.405 | 0.409 | 0.394 |
| Ala | 0.237 | 0.241 | 0.268 | 0.270 | 0.258 |
| Val | 0.026 | 0.027 | 0.025 | 0.024 | 0.025 |
| Ile | 0.007 | 0.007 | 0.006 | 0.007 | 0.007 |
| Leu | 0.007 | 0.007 | 0.006 | 0.006 | 0.006 |
| Tyr | 0.047 | 0.048 | 0.050 | 0.049 | 0.049 |
| Phe | 0.007 | 0.006 | 0.006 | 0.007 | 0.006 |
| His | 0.009 | 0.007 | 0.006 | 0.006 | 0.006 |
| Lys | 0.015 | 0.011 | 0.007 | 0.008 | 0.009 |
| Arg | 0.013 | 0.012 | 0.008 | 0.008 | 0.010 |

Silk dope dry weight mass


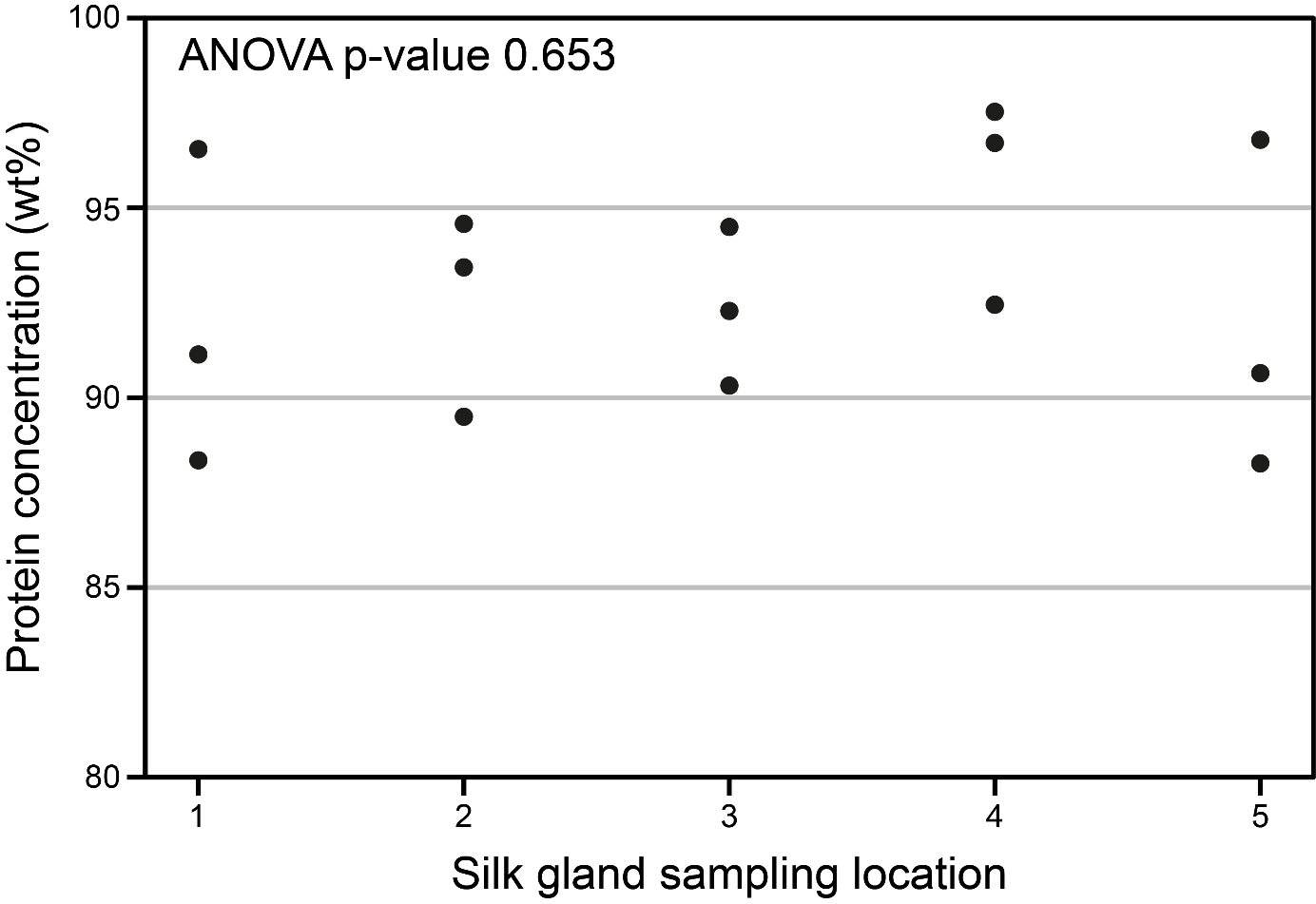


**Figure S8**. Protein content of dried silk dope samples. No significant correlation was found between the protein content and location in the MSG (ANOVA p-value 0.653).

**Extension of the silk dope with a motorized pulling setup**


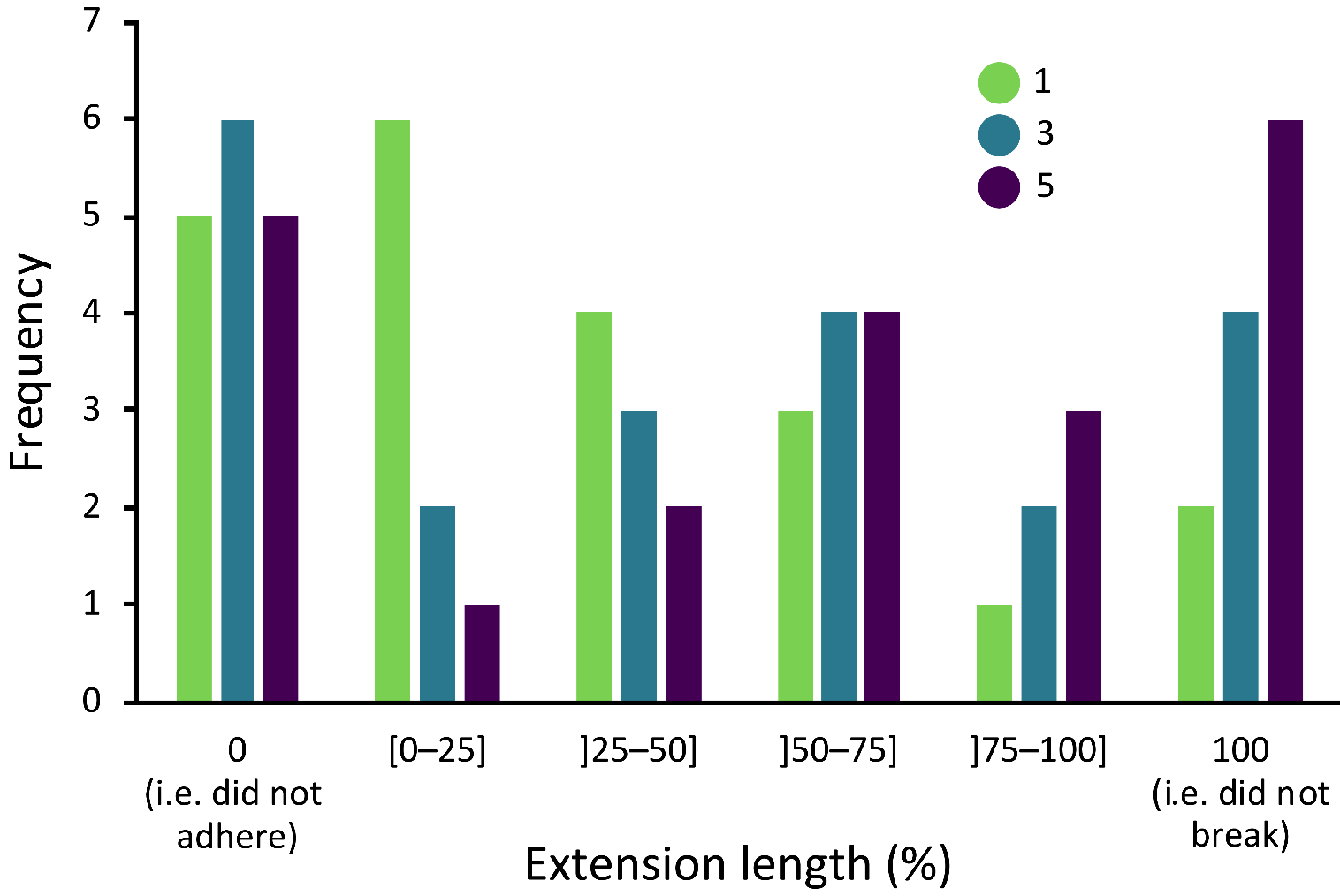


**Figure S9**. Extension of the silk dope from the posterior (1), middle (3), and anterior (5) region of the middle silk gland (MSG) with a motorized pulling setup. Pulling speed was set to ~0.5 mm/s, with maximum length of ~30 mm. Up to two samples of each region were taken per individual silkworm, and 21 samples were taken of each region. Occasionally, samples did not adhere to the metal plates of the pulling device, marked as “0”. The likelihood of a successful extension increased moving towards the end of the MSG. Note about the markings: ]25-50] equals to 25% or higher but less than 50% of the maximum extension length.

# References

[1] M. Andersson, J. Johansson, and A. Rising, “Silk Spinning in Silkworms and Spiders,” *Int. J. Mol. Sci.*, vol. 17, no. 8, p. 1290, Aug. 2016, doi: 10.3390/ijms17081290.

[2] X. Du, J. Li, and Y. Chen, “Proteomic analysis of sericin in Bombyx mori cocoons,” *Biotechnol. Bioprocess Eng.*, vol. 16, no. 3, pp. 438–444, Jun. 2011, doi: 10.1007/s12257-010-0425-0.

[3] W.-Q. Chen, H. Priewalder, J. P. Pradeep John, and G. Lubec, “Silk cocoon of *Bombyx mori* : Proteins and posttranslational modifications - heavy phosphorylation and evidence for lysine-mediated cross links,” *PROTEOMICS*, vol. 10, no. 3, pp. 369–379, Feb. 2010, doi: 10.1002/pmic.200900624.

[4] L. Lemetti, J. Tersteegen, J. Sammaljärvi, A. S. Aranko, and M. B. Linder, “Recombinant Spider Silk Protein and Delignified Wood Form a Strong Adhesive System,” *ACS Sustain. Chem. Eng.*, vol. 10, no. 1, pp. 552–561, Jan. 2022, doi: 10.1021/acssuschemeng.1c07043.

[5] S. Inoue, K. Tanaka, F. Arisaka, S. Kimura, K. Ohtomo, and S. Mizuno, “Silk Fibroin of Bombyx mori Is Secreted, Assembling a High Molecular Mass Elementary Unit Consisting of H-chain, L-chain, and P25, with a 6:6:1 Molar Ratio,” *J. Biol. Chem.*, vol. 275, no. 51, pp. 40517–40528, Dec. 2000, doi: 10.1074/jbc.M006897200.
